# The National Board of Health—The Medical Profession to the People of Savannah

**Published:** 1880-04

**Authors:** 


### THE NATIONAL BOARD OF HEALTH—THE MEDICAL PROFESSION TO THE PEOPLE OF SAVANNAH.

With pleasure we give place to the following protest against the proposed increase of unnecessary power:

In accordance with the terms of the accompanying protest, I beg to bring before you the action of the medical profession of Savannah upon the bills now pending before the Congress of the United States for the purpose of giving increased powers to the National Board of Health.

It is no part of the desire of the medical men of this city, as a body, to take sides with any of the theories concerning infectious diseases, nor do they accuse the members of the present National Board of Health or of the Executive Committee of any ulterior motive other than that of purely scientific investigations and sanitary good, when seeking the extraordinary powers proposed to be conferred upon them by these bills. The profession comes forward to warn the people of the dangerous and arbitrary powers which are sought to be conferred upon the National Board of Health under the specious plea of sanitary restrictions and quarantine regulations, because however good the composition of the Board may now be, however far above reproach, however distantly removed from anything like sectional proclivities or the influences of individual preferences, however free from the taint of bribery and corruption, the constitution of that Board may change. "Another Pharoah might arise," and then these powers might be employed to close your ports, ruin your commerce and beggar your people to the aggrandisement of more favored sections whose money power could make itself felt.

The medical profession is fully conscious that this warning should more properly have been sounded by the commercial portion of the country, by those whose interests would be most deeply affected by these enactments. But as this legislation is proposed upon the pretext of national health, and by a body of men whose duty it is to especially regard that health, the medical profession thinks it to be their duty to the public, as those to whom it is accustomed to look for information on such subjects, to warn it, that, in their opinion, the pretext is specious, and there exists no reason why such legislation should be had; in order that if the public consents thereto, its consent may be given with full knowledge of the facts, and not under a false impression as to its necessity. They, therefore, suggest a convention of all the people interested, that a proper decision may be arrived at.

The Nashville Board of Health has already sent the protest in the following language:

* * * "We * * * would inform you" (Senators and Representatives) "that it is the sense of our Board that the contemplated amendment is highly inexpedient, and it is calculated to inflict serious detriment to the commercial interest of a city without compensating good. It would place the decision of a special case of disease in the hands of those who are at least foreign to the local welfare and prosperity, ignorant of the antecedents and surroundings. Furthermore, the enactment of such a law would give occasion to a conflict of opinion and authority in matters strictly medical, thereby retarding the progress of sanitary work rather than fostering and encouraging it?'

The State Board of Health of South Carolina has also protested, and Darien and other cities have raised their voice against it, and from letters received, both North and South, it is understood that the appointment of delegates is only delayed until the time and place for holding the convention be appointed. R. J. NUNN, M.D.

Savannah, Ga., April 6, 1880.

At a meeting of the Georgia Medical Society of Savannah, held March 23, 1880, the bills now before Congress to increase the power of the National Board of Health were discussed, and as it is contrary to the by-laws of the society to pass resolutions upon matters of a public nature, it was decided that a paper be prepared expressive of the views of the individual members, to be signed by them and presented to the City Council, Cotton Exchange, and the people of Savannah. In accordance with these instructions, the following is offered:

We, the undersigned, members of the Georgia Medical Society, view with apprehension the extraordinary and dangerous powers proposed to be conferred upon the National Board of Health by the bills introduced in Congress by Hon. Messrs. Harris and Young, and also that proposed by Mr. Acklen. The first section of Mr. Harris' bill provides "That the National Board of Health, or in the interval of its sessions, its Executive Committee (of five) shall report to the President of the United States, whenever any place in the United States is considered by it to be dangerously infected with contagious or infectious disease, and that upon the official publication by the President of such report, the transportation of goods or persons from such place into another State shall be unlawful, and all persons guilty thereof shall be liable to prosecution therefor in the Circuit or District Court of the United States for any district within which such goods or persons shall be transported, and any goods so transported shall be liable to be seized and destroyed, unless such transportation shall be carried on in accordance with rules and regulations made by the National Board of Health and approved by the President as in other cases. These rules shall apply until the President shall proclaim such place no longer dangerously infected, and in the meantime the Board, or its Executive Committee, shall report to him weekly, in writing, the sanitary condition of the place in question."

Mr. Acklen's bill provides for the establishment of quarantine stations upon all the avenues of approach to any place declared to be infected, upon information furnished by the National Board of Health, at which points all freights or persons and their vehicles of transportation, if starting from or destined for a point beyond the borders of the State, shall be inspected by the agents of the National Board of Health, and after they shall have been declared by such agents free from infection, any person or authority whatsoever who shall interfere with the free passage of such person, freight or vehicle of transportation, on the charge that they are infected or dangerous to the public health, shall, on conviction before a United States Court, be fined one thousand dollars and imprisonment one year.

We consider the provisions of these bills as dangerous, revolutionary and useless should they become laws. We protest most strenuously against conferring such extraordinary powers upon any National Board of Health, believing that such powers could be used to destroy our commerce and liberties, and are subversive of the rights of States.

We earnestly appeal to the municipal authorities, the merchants and the public of Savannah to unite in a protest against the enactment of such laws. We would further suggest the holding of a convention, in which all of the Atlantic and Gulf States should be requested to participate, in order to frame a bill wherein our common weal should be explicitly provided for.

| | |
|---|---|
| R. J. Nunn, M.D., | W. Duncan, M.D. |
| Pres. Ga. Med. Soc. | Benj. F. Sheftall, M.D. |
| Jas. B. Read, M.D., | Jno. D. Martin, M.D. |
| Henry Le Hardy, M.D., | J. S. Morel, M.D. |
| Sec., Treas. and Lib. G.M.S. | Frank T. Lincoln, M.D. |
| Jno. M. Johnston, M.D., | J. C. Le Hardy, M.D. |
| Cor. Sec'y G. M. S. | J. T. McFarland, M.D. |
| J. P. S. Houstoun, M.D. | Wm. G. Bulloch, M.D. |

T. B. Chisholm, M.D.

Robt. P. Myers, M.D.

T. J. Charlton, M.D.

Benj. S. Purse, M.D.

H. Burford, M.D.

B. W. Hardee, M.D.

B. S. Herndon, M.D.

Three members dissenting, one not replying.

Correspondence to be sent to Dr. J. C. LeHardy, Savannah, Ga.

OFFICE OF THE PRESIDENT OF THE GEORGIA MEDICAL SOCIETY.
SAVANNAH, GA., March 27, 1880.

The above is a complete roll of the members of the Georgia Medical Society at present in the city of Savannnah.

R. J. NUNN, M.D.
President Georgia Medical Society.

FRANK T. LINCOLN, M.D., Sec. pro. tem. Ga. M. S.

We, the undersigned physicians, practicing in the city of Savannah, concur in the sentiments embodied in the above protest.

C. C. Schley, M.D.,

Eugene Rollin Carson, M.D.,

J. Weischselbaum, M.D.,

L. A. Falligant, M.D.

Louis Knorr, M.D.

A. B. George, M.D.,

R. S. Sanders, M.D.

The undersigned, members of the Savannah Cotton Exchange, fully concur in the sentiments expressed in the foregoing protest.

J. H. Johnston,

Vice Pre. Sav. Cot. Ex.

Octavus Cohen & Co.,

M. Hamilton,

Holst, Fullarton & Co,,

L. J. Guilmartin,

Chas. C. Hardwick,

T. D. Bertody,

P. D. Daffin,

Jas. B. West,

Jas. T. Stewart,

H. P. Richmond,

D. Y. Dancy,

A. Minis & Son,

J. McMahon,

Vice Pres. South'n Bank.

B. B. Minor, Jr.,

Jos. B. Duckworth,

Charles Green & Co.,

H. Kuhn,

John Flannery & Co.,

C. H. & W. H. Way,

Walter & Hart,

Hartley & Russell,

Gouldin, Young & Frost,

JOHN F. WHEATON,

Mayor City of Savannah.

L. C. LEHARDY, Savan'h, Ga.

